# Analgesic use in a Norwegian general population: change over time and high-risk use - The Tromsø Study

**DOI:** 10.1186/s40360-015-0016-y

**Published:** 2015-06-06

**Authors:** Per-Jostein Samuelsen, Lars Slørdal, Ulla Dorte Mathisen, Anne Elise Eggen

**Affiliations:** 1 Regional Medicines Information and Pharmacovigilance Center (RELIS), University Hospital of North Norway, N-9038 Tromsø, Norway; 2 Department of Community Medicine, UiT-The Arctic University of Norway, Tromsø, Norway; 3 Department of Laboratory Medicine, Children’s and Women’s Health, Norwegian University of Science and Technology, Trondheim, Norway; 4 Department of Clinical Pharmacology, St. Olav University Hospital, Trondheim, Norway; 5 Section of Nephrology, University Hospital of North Norway, Tromsø, Norway

**Keywords:** Analgesics, NSAIDs, Drug interactions, Contraindications, Prevalence, Pharmacoepidemiology

## Abstract

**Background:**

Increased use of analgesics in the population is a cause for concern in terms of drug safety. There is a paucity of population-based studies monitoring the change in use over time of both non-prescription (OTC) analgesics and prescription (Rx) analgesics. Although much is known about the risks associated with analgesic use, we are lacking knowledge on high-risk use at a population level. The purpose of this study was to estimate the prevalence of non-prescription and prescription analgesic use, change over time and the prevalence in the presence of potential contraindications and drug interactions in a general population.

**Methods:**

A repeated cross-sectional study with data from participants (30–89 years) of the Tromsø Study in 2001–02 (Tromsø 5; *N* = 8039) and in 2007–08 (Tromsø 6; *N* = 12,981). Participants reported use of OTC and Rx analgesics and regular use of all drugs in the preceding four weeks. Change over the time period was analyzed with generalized estimating equations. The prevalence of regular analgesic use in persons with or without a clinically significant contraindication or drug interaction was determined in the Tromsø 6 population, and differences were tested with logistic regression.

**Results:**

Analgesic use increased from 54 to 60 % in women (OR = 1.24, 95 % CI 1.15–1.32) and from 29 to 37 % in men (OR = 1.39, 95 % CI 1.27–1.52) in the time period; the increase was due to sporadic use of OTC analgesics. There was substantial regular use of analgesics in several of the contraindication categories examined; the prevalence of non-steroidal anti-inflammatory drugs was more than eight per cent among persons with chronic kidney disease, gastrointestinal ulcers, or high primary cardiovascular risk. About four per cent of the study population demonstrated at least one potential drug interaction with an analgesic drug.

**Conclusions:**

The use of analgesics increased in the time period due to an increase in the use of OTC analgesics. Analgesic exposure in the presence of contraindications or drug interactions may put patients at risk. Public and prescriber awareness about clinically relevant contraindications and drug interactions with analgesics need to be increased.

## Background

The availability of analgesics has increased in Norway due to a major rise in the number of pharmacies since 2001 and the release of ibuprofen and paracetamol to general sales in supermarkets, grocery stores and petrol stations in 2003 [1]. The sales of analgesics has increased considerably in Norway over the last decades [2]. The prevalence of analgesic use has been examined in several international cross-sectional studies [3–10]. However, there is a paucity of population-based studies monitoring the change in use over time of both OTC analgesics and prescription (Rx) analgesics.

Increased use of analgesics in the population is a cause for concern in terms of drug safety. Paracetamol and in particular non-steroidal anti-inflammatory drugs (NSAIDs) and opioids are among the drugs that are most often implicated in serious or fatal medication errors [11]. NSAID use is associated with increased risk of cardiovascular disease (CVD) [12–14], gastrointestinal damage [15], renal disease [16, 17] and a number of drug interactions [15, 18]. Opioids have an abuse potential but few adverse effects when used correctly. They can, however, produce respiratory depression [15, 19] and increase the risk of falls and subsequent injuries [20], particularly in combinations with other central nervous system (CNS) depressant drugs [19]. Paracetamol, although considered safe in recommended doses, is hepatotoxic in high doses and can give rise to drug induced liver injury [21]; there is some concern about a possible association with increased CVD risk [22]. The potential inappropriate use of analgesics in the general population has previously been reported in smaller studies of variable rigor, most of them focusing on OTC analgesics [7, 23–27].

This study aimed to estimate the prevalence of OTC and Rx analgesic use, change over time and the prevalence of use in the presence of potential contraindications and pharmacodynamic drug interactions.

## Methods

### Study population

**Fig. 1:**
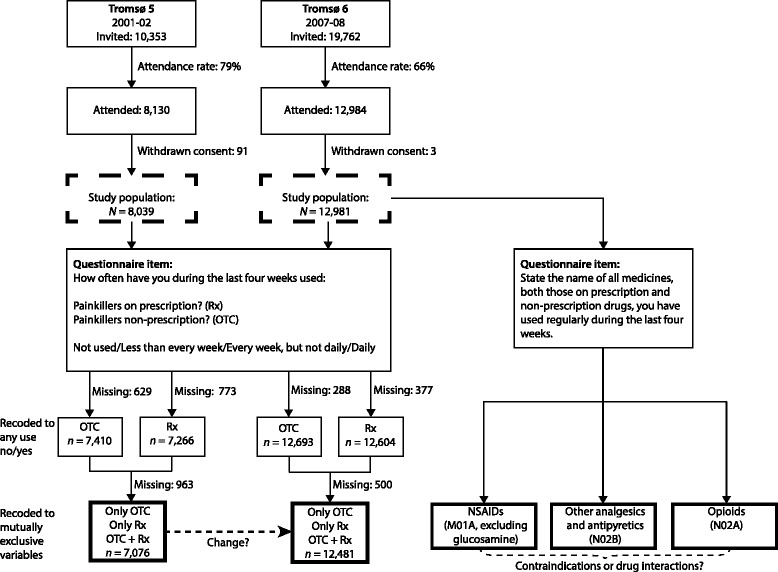
Flow chart of the study and questionnaire items. OTC = “over-the-counter”, non-prescription; Rx = prescription; NSAIDs = non-steroidal anti-inflammatory drugs

The Tromsø Study is a population-based study of various health issues and diseases. It consists of six surveys (Tromsø 1–6) carried out in the municipality of Tromsø, Norway, from 1974 to 2008 [28]. Eligible for the present study were participants from Tromsø 5 (2001–02, *N* = 8039) and Tromsø 6 (2007–08, *N* = 12,981), aged 30–89 years (Fig. 1). The participants in Tromsø 5 and Tromsø 6 consisted of persons who attended a second visit in Tromsø 4 in 1994–5 (i.e. all Tromsø inhabitants aged 55–74 years, and a 5–10 % random sample of those 25–50 and 75–85 years of age were invited), in addition to whole birth cohorts or random samples of birth cohorts (see [28, 29] for further explanation). A total of 4630 individuals participated in both Tromsø 5 and Tromsø 6. The data collection is described elsewhere [29]. An English translation of the questionnaires is available at the Tromsø Study homepage (www.tromsostudy.com).

### Definition of analgesic use

Analgesic use was assessed through questionnaire based on the question “How often have you used painkillers [with]/[without] prescription during the last four weeks?” (Fig. 1). Analgesic users were defined as persons reporting any use. This variable was recoded into use of OTC analgesics only (“OTC”), use of prescribed analgesics only (“Rx”) and use of concomitantly OTC and prescribed analgesics (“OTC + Rx”; Fig. 1).

Participants in Tromsø 6 also reported drugs used regularly the preceding four weeks; this was coded according to the Anatomical Therapeutic Chemical (ATC) classification system version 2007 (www.whocc.no). Analgesics were defined as belonging to ATC groups N02B (other antipyretic and analgesic drugs), N02A (opioids) and M01A (NSAIDs, excluding glucosamine).

### Criteria for contraindications and drug interactions

The analyses of potential contraindications and drug interactions were done among participants in Tromsø 6 (Fig. 1). A contraindication was defined as a condition that indicates that a drug should not be used. The criteria were developed a priori, based on literature and available variables:

*Chronic kidney disease*: estimated glomerular filtration rate (eGFR) < 60 ml/min per 1.73 m^2^ or ≥ 60 ml/min per 1.73 m^2^ and either macroalbuminuria or persistent microalbuminuria [30]. EGFR was estimated by the CKD-EPI equation [31]. *Gastrointestinal ulcers*: self-reported stomach or duodenal ulcer or ulcer surgery. A secondary measure was use of H2 antagonists, misoprostol or proton pump inhibitors (ATC codes A02B A, A02B B, A02B C, respectively). *CVD*: NORRISK cardiovascular risk score estimates the 10-year risk of fatal CVD, using sex, age, systolic blood pressure, total cholesterol and smoking [32]. The primary CVD risk group was defined as individuals with no prior myocardial infarction (MI), angina pectoris or stroke, aged 40–49 years and with a NORRISK score > 1 %; 50–59 years and NORRISK score ≥ 5 %; or 60–69 years and NORRISK score ≥ 10 % according to national guidelines [33]. The secondary CVD risk group consisted of those with a history of stroke, MI or angina pectoris. *Hypertension*: systolic blood pressure ≥ 140 mmHg or diastolic blood pressure ≥ 90 mmHg or self-reported current use of antihypertensive drugs. *Interacting drugs:* warfarin (B01A A03), low-dose acetylsalicylic acid (ASA; B01A C06), selective serotonin reuptake inhibitors (SSRI; N06A B), glucocorticoids (H02A B), angiotensin converting enzyme (ACE) inhibitors (C09A, C09B), angiotensin II (AT II) antagonists (C09C, C09D), other antihypertensive drugs (C02, C03, C07, C08) and CNS depressant drugs (N05C A-F, N05B A, N03A E, N03A A). *Use of multiple analgesics*: regular use of more than one analgesic drug within the same pharmacological group: NSAIDs, opioids and paracetamol-containing drugs (N02B E01 and N02A A59).

### Statistical analysis

Descriptive statistics were age-adjusted with logistic regression (*adjprop* command). The changes in prevalences between Tromsø 5 and Tromsø 6 were tested with generalized estimating equations (GEE) and estimated as odds ratios (ORs) with 95 % confidence intervals (CI) using a logit link function, exchangeable covariance matrix and robust standard errors; separate binary GEE models were fitted for each prescription category with non-users of both OTC and Rx as the reference group. The prevalence measures were age-adjusted by the direct method, with the Norwegian population per 01.01.2008 as standard population [34]. Linear age trends across age groups were tested with logistic regression. Sex differences in age-adjusted prevalences were tested with two-sample proportion test (Z test) and crude prevalences with Fisher’s exact test. Differences in analgesic use in the absence or presence of contraindications or drug interactions were tested with logistic regression and likelihood ratio test, adjusted for age and sex (*adjprop*). All analyses were complete case analyses. The overall proportion of missing data in the dependent variables in the GEE analyses was 12.0 % in Tromsø 5 and 3.9 % in Tromsø 6 (Fig. 1). Sensitivity analyses by imputing missing values as non-user or user, were generally consistent with the main results. All analyses were conducted in Stata 13.1 (Stata Corp, College Station, Texas).

### Ethics

This study has been approved by the Regional Committee for Medical and Health Research Ethics, North Norway (2012/1636), and was performed in accordance with the 1964 Helsinki declaration and its later amendments. Informed consent was obtained from all individual participants included in the study.

## Results

There was a tendency of worsening health, more pain and less education across the analgesic user groups, from non-users to users of OTC + Rx analgesics (Table 1).

**Table 1:** Charactheristics of non-users and users of OTC, Rx, or combined OTC + Rx analgesics

	Non-users		OTC		Rx		OTC + Rx	
	% (*n*)		% (*n*)		% (*n*)		% (*n*)	
Analgesic use (*n* = 12,481)^a^	53.8	(6719)	31.7	(3957)	5.1	(641)	9.3	(1164)
Sex, % women (*n* = 12,481)	41.8	(2818)	65.1	(2561)	58.6	(378)	70.8	(824)
Age (*n* = 12,481)								
30–39	3.2	(213)	6.2	(247)	1.2	(8)	3.3	(38)
40–49	23.1	(1549)	37.4	(1479)	20.7	(133)	31.4	(365)
50–59	18.4	(1236)	20.5	(813)	16.8	(108)	18.1	(211)
60–69	35.5	(2388)	24.7	(979)	35.7	(229)	28.4	(330)
70–79	15.5	(1041)	8.8	(347)	20.1	(129)	14.8	(172)
80–87	4.3	(292)	2.3	(92)	5.3	(34)	4.1	(48)
Mean (SD), 30–87 years	58.9	(12.3)	53.6	(12.3)	60.8	(12.0)	57.1	(12.8)
Bad or very bad self-reported health (*n* = 12,390)	3.2	(226)	4.3	(162)	10.1	(70)	15.9	(185)
Education below college or university (*n* = 12,341)	59.9	(4029)	63.0	(2278)	69.4	(451)	74.3	(827)
Smoking, current daily (*n* = 12,329)	18.1	(1194)	21.1	(873)	24.1	(146)	27.5	(318)
Pain lasting three months or more (*n* = 12,462)	19.9	(1344)	35.6	(1393)	63.1	(404)	74.7	(868)
Headache, last year (*n* = 11,472)	18.5	(1156)	50.0	(1928)	46.7	(257)	61.3	(645)
Severe pain or stiffness in muscles, last four weeks								
Neck (*n* = 10,665)	4.2	(243)	9.6	(326)	20.1	(104)	29.5	(289)
Hip/leg (*n* = 10,531)	3.8	(237)	7.8	(249)	20.5	(122)	28.9	(280)
Psychological distress^b^ (*n* = 11,941)	5.4	(343)	8.7	(340)	11.4	(67)	20.9	(230)
Frequent GP consultations, last 12 months^c^ (*n* = 9335)	10.9	(538)	11.6	(343)	19.7	(105)	25.2	(243)
Drug use, last four weeks								
Antidepressants (*n* = 12,195)	1.6	(116)	2.9	(106)	4.3	(29)	9.0	(97)
Sleeping pills or tranquilizers (*n* = 12,164)	5.7	(476)	12.0	(445)	14.8	(112)	27.3	(297)

Age-adjusted. The Tromsø Study: Tromsø 6 (2007–8, *n* = 12,481)

*OTC* “over-the-counter”, non-prescription, *Rx* prescription, *SD* standard deviation, *GP* general practitioner

^a^Crude prevalence

^b^Hopkins Symptoms Checklist 10-item version > 1.85

^c^≥ 6 visits per year (>90th percentile)

Women used more analgesics than men, both in total and in all prescription categories, in both surveys (Table 2). The total analgesic use decreased with age in both sexes and in both surveys (*p* < .001). The use of OTC decreased, whereas Rx increased with age in both sexes and in both surveys.

**Table 2:** Prevalence of analgesic use and change over time

	Population		OTC only			Rx only			OTC + Rx			Total		
Survey	T5	T6	T5	T6		T5	T6		T5	T6		T5	T6	
Age (years)	*n* (%)	*n* (%)	%	%	OR (95 % CI)^a^	%	%	OR (95 % CI)^a^	%	%	OR (95 % CI)^a^	%	%	OR (95 % CI)^a^
Women														
30–39	408 (10.4)	295 (4.5)	46.3	55.6	1.45 (1.05–1.99)	2.2	1.4	0.75 (0.23–2.48)	10.3	9.2	1.09 (0.63–1.86)	58.8	66.1	1.37 (1.00–1.86)
40–49	710 (18.1)	1880 (28.6)	44.9	49.3	1.29 (1.07–1.56)	5.2	3.8	0.87 (0.57–1.31)	10.9	13.6	1.41 (1.08–1.84)	61.0	66.6	1.29 (1.08–1.53)
50–59	637 (16.2)	1245 (18.9)	32.2	43.6	1.68 (1.36–2.07)	6.0	5.1	1.05 (0.68–1.61)	12.7	11.7	1.85 (0.71–4.80)	50.9	60.4	1.49 (1.23–1.79)
60–69	1187 (30.2)	1987 (30.2)	29.0	31.4	1.11 (0.96–1.29)	7.6	7.1	0.96 (0.74–1.24)	11.2	11.5	1.01 (0.83–1.22)	47.8	50.0	1.06 (0.94–1.21)
70–79	871 (22.2)	886 (13.5)	23.7	26.1	1.16 (0.94–1.43)	9.5	8.5	0.95 (0.69–1.31)	11.5	14.3	1.25 (1.00–1.56)	44.7	48.9	1.14 (0.96–1.36)
80+	118 (3.0)	288 (4.4)	25.4	25.7	1.15 (0.68–1.92)	5.9	8.3	1.18 (0.80–1.77)	15.3	13.9	0.98 (0.53–1.82)	46.6	47.9	1.15 (0.76–1.74)
30–89	3931 (100)	6581 (100)	32.9	38.9	1.26 (1.17–1.36)	6.7	5.7	0.98 (0.84–1.14)	11.5	12.5	1.19 (1.08–1.30)	51.1	57.2	1.21 (1.13–1.30)
Age–adjusted^b^			36.7	42.9	1.30 (1.20–1.41)	5.5	4.8	0.98 (0.84–1.14)	11.5	11.9	1.20 (1.09–1.32)	53.7	59.6	1.24 (1.15–1.32)
* p*, age trend			<.001	<.001		<.001	<.001		.370	.471		<.001	<.001	
Men														
30–39	273 (8.7)	211 (3.6)	30.4	39.3	1.58 (1.08–2.30)	1.8	1.9	1.23 (0.32–4.64)	4.4	5.2	1.40 (0.60–3.29)	36.6	46.5	1.55 (1.08–2.22)
40–49	569 (18.1)	1646 (27.9)	27.2	33.6	1.45 (1.18–1.78)	2.6	3.8	1.69 (0.97–2.95)	3.3	6.7	2.39 (1.45–3.94)	33.2	44.1	1.56 (1.29–1.89)
50–59	332 (10.6)	1123 (19.0)	18.4	24.0	1.34 (1.01–1.79)	6.3	4.0	0.68 (0.42–1.13)	2.4	5.8	2.63 (1.25–5.51)	27.1	33.8	1.29 (1.01–1.65)
60–69	1108 (35.2)	1939 (32.9)	14.0	18.4	1.37 (1.13–1.66)	5.7	4.5	0.84 (0.61–1.15)	4.5	5.2	1.19 (0.87–1.63)	24.2	28.1	1.21 (1.04–1.41)
70–79	769 (24.5)	803 (13.6)	10.4	14.5	1.52 (1.13–2.06)	5.6	6.7	1.31 (0.88–1.94)	4.3	5.6	1.43 (0.91–2.24)	20.3	26.8	1.46 (1.17–1.83)
80+	94 (3.0)	178 (3.0)	10.6	10.1	0.93 (0.42–2.05)	7.5	5.6	0.75 (0.28–2.05)	1.1	4.5	4.27 (0.53–34.60)	19.2	20.2	1.05 (0.56–1.96)
30–89	3145 (100)	5900 (100)	17.3	23.7	1.46 (1.32–1.61)	4.9	4.5	1.02 (0.84–1.23)	3.9	5.8	1.59 (1.31–1.92)	26.1	33.9	1.38 (1.27–1.50)
Age–adjusted^b^			21.3	27.1	1.48 (1.33–1.65)	4.3	3.9	1.03 (0.85–1.24)	3.5	5.7	1.57 (1.29–1.91)	29.1	36.7	1.39 (1.27–1.52)
* p*, age trend			<.001	<.001		.001	.001		.727	.127		<.001	<.001	
*p*, sex			<.001	<.001		.037	.046		<.001	<.001		<.001	<.001	

The proportion of analgesic users last four weeks and odds ratios for use of analgesics in Tromsø 6 compared to Tromsø 5, according to age, sex and prescription category. The Tromsø Study: Tromsø 5 (2001–02, *n* = 7076) and Tromsø 6 (2007–08, *n* = 12,481)

*OTC* “over-the-counter”, non-prescription, *Rx* prescription, *T5* Tromsø 5, *T6* Tromsø 6, *CI* confidence interval, *OR *odds ratio

^a^Reference category: non-users of both OTC and Rx

^b^Prevalence estimates age-adjusted with the Norwegian population 01.01.2008 as standard population

The total use of analgesics and the use of OTC increased in the time period (Table 2). Total use increased from 53.7 to 59.6 % in women and from 29.1 to 36.7 % in men, corresponding to OR = 1.24, 95 % CI 1.15–1.32 and OR = 1.39, 95 % CI 1.27–1.52, respectively. The use of Rx analgesics did not show any change, while the use of OTC + Rx analgesics increased in both women and men. When the analyses were restricted to frequent users, defined as daily or weekly users, there was no change in total use (data not shown).

**Fig. 2:**
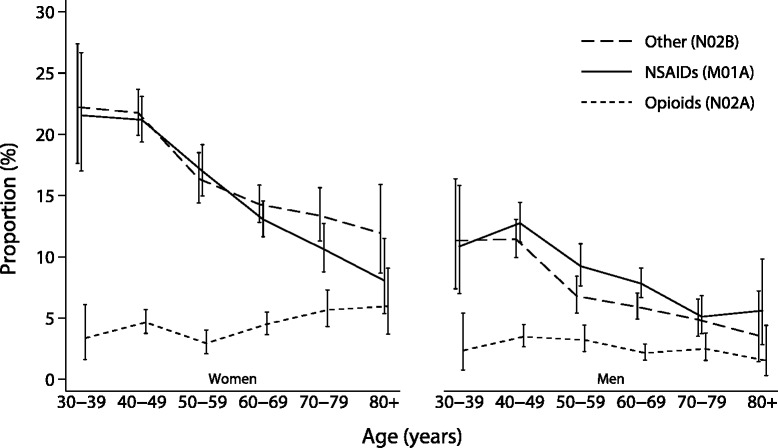
Regular analgesic use, both non-prescription and prescribed, last four weeks according to age and sex. The Tromsø Study: Tromsø 6 (*N* = 12 981). Other analgesics and antipyretics (long dashed line), non-steroidal anti-inflammatory drugs (solid line) and opioids (short dashed line). Vertical lines are the 95 % confidence intervals. “Other” include paracetamol (which constitutes over 95 %), high-dose acetylsalicylic acid and phenazone-caffeine

The crude prevalences of regular use of NSAIDs, other analgesics and antipyretics, and opioids were 12.7 % (*n* = 1646), 12.5 % (*n* = 1624) and 3.7 % (*n* = 475), respectively. The prevalences of cyclooxygenase 2 (COX-2) inhibitors and high-dose ASA use were 0.1 % (*n* = 13) and 0.2 % (*n* = 31), respectively. The use of NSAIDs and other analgesics decreased with age in both sexes (*p* < .001), while opioid use increased in women (*p* = .048) and decreased in men (*p* = .027; Fig. 2). More women than men used analgesics regularly (*p* < .001). The sex difference for opioids was only apparent in the highest age groups (≥60 years).

Table 3 shows the prevalence of regular analgesic use in the absence or presence of contraindications. The prevalence was high in several of the contraindication groups; for the important contraindications chronic kidney disease, gastrointestinal ulcer diseases and high primary cardiovascular risk there were no differences in regular NSAID use between those with and without the contraindication, when adjusting for age and sex differences. Among the categories examined, only persons with a history of CVD had a lower prevalence of NSAID use compared to those without a CVD history.

**Table 3:** Regular use of analgesics in the absence or presence of contraindications

	Unadjusted		Age- and sex-adjusted			
Contraindication^a^	Absent	Present	Absent	Present	*p* value	Potential clinical consequence
	% (*n*)	% (*n*)	%	%		
*Non-steroidal anti-inflammatory drugs*						
Chronic kidney disease (6834/10.1)	11.2 (686)	8.6 (59)	11.6	12.0	.802	Acute renal failure, disease progression
GI ulcers						
Ulcers (11,516/7.4)	12.8 (1365)	12.0 (102)	11.8	12.6	.509	GI ulceration and complications
Ulcers or use of GI-protective drugs (11,516/10.7)	12.7 (1301)	13.4 (166)	11.6	14.1	.014	GI ulceration and complications
CVD						
High primary CVD risk (9000/13.0)	14.4 (1125)	11.1 (129)	12.1	13.5	.220	Increased risk of CVD
Stroke, MI, angina pectoris (12,540/9.6)	13.3 (1506)	6.7 (80)	12.1	8.8	.003	Increased risk of CVD
Hypertension (12,725/49.1)	14.2 (922)	11.3 (705)	11.5	12.5	.122	Increased blood pressure
*Paracetamol*						
CVD						
High primary CVD risk (9000/13.0)	14.5 (1139)	9.7 (113)	11.6	12.6	.401	Possible increased risk of CVD
Stroke, MI, angina pectoris (12,540/9.6)	13.8 (1565)	9.9 (119)	12.3	12.8	.712	Possible increased risk of CVD

The Tromsø Study: Tromsø 6 (*N* = 12,981)

*GI* gastrointestinal, *CVD* cardiovascular disease, *MI* myocardial infarction

^a^Numbers in parentheses are total *n* in variable and prevalence (%) in the study population

Four hundred and sixteen instances of use of multiple analgesics were found in 384 persons; the proportions were 11.2 % (*n* = 184) in NSAID users, 12.0 % (*n* = 209) in users of paracetamol-containing analgesics and 4.8 % (*n* = 23) in opioid users.

Table 4 shows the prevalence of regular analgesic use in the absence or presence of interacting drugs. In total 4.1 % (*n* = 538) of the population presented at least one of the identified potential drug interactions. One percent presented more than one potential drug interaction. For interactions potentially increasing the bleeding risk, the use of NSAIDs was the same or higher among users of glucocorticoids or SSRIs, respectively. The use of NSAIDs was comparatively lower for patients using the anticoagulant warfarin and low-dose ASA.

**Table 4:** Regular use of analgesics in the absence or presence of interacting drugs

	Unadjusted		Age- and sex-adjusted			
Interacting drug^a^	Absent	Present	Absent	Present	*p* value	Potential clinical consequence
	% (*n*)	% (*n*)	%	%		
*Non-steroidal anti-inflammatory drugs*						
Warfarin (2.5)	12.9 (1637)	2.8 (9)	12.1	3.9	<.001	Increased bleeding risk
ASA, low dose (11.7)	13.6 (1558)	5.8 (88)	12.5	7.4	<.001	Increased bleeding risk
SSRI (1.5)	12.5 (1602)	22.0 (44)	11.8	19.5	.001	Increased bleeding risk
Glucocorticoids (1.3)	12.7 (1625)	12.6 (21)	11.9	13.0	.672	Increased bleeding risk
ACE inhibitors (3.8)	12.9 (1614)	6.5 (32)	12.0	7.8	.007	Diminished effect, renal impairment, hyperkalemia
AT II antagonists (9.2)	12.8 (1508)	11.5 (138)	11.8	12.8	.326	Diminished effect, renal impairment, hyperkalemia
Other antihypertensives (18.8)^b^	13.3 (1397)	10.2 (249)	11.8	12.1	.693	Diminished effect
*Opioids*						
CNS depressants (4.9)^c^	2.9 (359)	18.1 (116)	2.9	17.5	<.001	CNS depression, respiratory depression, falls
*Paracetamol*						
Warfarin (2.5)	13.6 (1719)	7.1 (23)	12.5	9.5	.154	Increased bleeding risk

The Tromsø Study: Tromsø 6 (*N* = 12,981)

*ASA* acetylsalicylic acid, *SSRI* selective serotonin reuptake inhibitors, *ACE* angiotensin converting enzyme, *AT II* angiotensin II, *CNS* central nervous system

^a^The number in parentheses is the prevalence (%) in the study population

^b^ATC-groups C02, C03, C07, C08

^c^Benzodiazepines, z hypnotics and barbiturates (ATC-groups N05C A-F, N05B A, N03A E, N03A A)

## Discussion

The use of analgesics increased from 2001–02 to 2007–08, due to an increase in the use of OTC analgesics. The prevalence of regular analgesic use in the contraindication categories examined was more than six per cent, and about four per cent of the study population presented at least one potential drug interaction with an analgesic drug. In particular, the use of NSAIDs in the presence of chronic kidney disease, gastrointestinal ulcers, high primary risk of CVD and interacting drugs increasing the bleeding risk was a cause for concern.

The sales of NSAIDs more than doubled, paracetamol tripled, while high-dose ASA declined substantially in Norway from 1990 to 2013 [2]. A comparison of our data with data from the 1980s and 1990s [3–5, 10], and studies on changes in Rx analgesic use [35–37] points towards an increase in analgesic use from the 1980s to the present. However, a US study employing data from around 1990 shows much higher use of OTC analgesics in corresponding age groups compared to our findings, whereas the use of Rx analgesics was lower in women and comparable in men [6], suggestive of a different usage pattern in the US. We found no increase in frequent analgesic use in the time period, reflecting that the increase was due to sporadic use of OTC analgesics. Possible hypotheses for the trend in analgesic use include increased prevalence of pain, a shift in the attitude towards perceived pain and/or drug use, and increased availability. It has been previously shown that a switch to OTC status leads to an initial increase in total sales of the drug [38], while the release to general sales may increase the use of NSAIDs [26]. However, the possible link between increased availability and increased use warrants further research.

Users of OTC NSAIDs are generally unaware of or unconcerned with the potential harmful effects, as OTC drugs are perceived to be relatively safe [27]. The recommended doses of OTC NSAIDs are lower than the recommended prescription doses. However, use of OTC analgesics in doses exceeding the maximum has been reported [27].

For most of the contraindications examined, the prevalence of analgesic use was not different between persons with and without the condition when adjusted for age and sex differences. This suggests lack of awareness about the contraindications. We demonstrated frequent use of multiple analgesics within the same pharmacological group, in line with previous studies [25, 26], and frequent combined use of OTC and Rx analgesics; this would be expected to increase the risk of dose-dependent adverse effects.

NSAID use is associated with further renal impairment in individuals with underlying kidney disease [15], acute renal failure [16] and progression of chronic kidney disease [17]. We found no difference in regular use of NSAIDs among those with and without chronic kidney disease, in line with previous research [39]. Some eight percent (*n* = 59) of subjects reported using NSAIDs regularly despite having chronic kidney disease, putting them at risk of disease progression and acute renal failure.

The use of NSAIDs was not affected by a history of gastrointestinal ulcers. However, the prevalence of NSAID use was higher in patients using gastroprotective agents than in non-users. The use of gastroprotective agents may be considered either as a marker of gastrointestinal disease, i.e. a risk factor, or as a prudent precautionary measure, i.e. the prophylactic use of gastroprotective agents in persons at increased risk.

The prevalence of NSAID use was lower in persons with a history of CVD compared with those with no CVD. The risk of CVD is increased by most NSAIDs, even as short-term treatment and both in healthy individuals and in those with known CVD [12, 14] or high CVD risk [13]. There were almost no users of COX-2 inhibitors in our study population. However, diclofenac is comparable to the COX-2 inhibitors in terms of CVD risk [12]. Diclofenac is the second most sold NSAID in Norway [2] and has been available as an OTC drug from 2001.

If our data are applied to the entire Norwegian population per 2008 [34], approximately 25,000 persons aged 40–69 years with high primary CVD risk would be regular NSAID users. The NORRISK equation overestimates the prevalence of high primary CVD risk, but nevertheless our results suggest that being at high primary CVD risk does not lead to lower NSAID use. This constitutes an important problem even when the absolute risks in the population decrease.

Low-dose ASA, glucocorticoids, SSRIs, and in particular warfarin increase the gastrointestinal bleeding risk when combined with NSAIDs [15]. The much lower prevalence of NSAID use among warfarin users may be due to prescriber diligence and frequent consultations with this group of patients. Our results do however suggest that the concomitant use of glucocorticoids or SSRIs with NSAIDs is not perceived as problematic.

The prevalence of NSAID use was lower among patients taking ACE inhibitors compared to non-users, while no such difference was found for the AT II antagonists. This is somewhat surprising, as the combination of either ACE inhibitors or AT II antagonists with NSAIDs is associated with both diminished antihypertensive efficacy as well as an increased risk of renal impairment and hyperkalemia [18].

Our results show a high degree of co-medication with CNS depressant drugs among opioid users, in agreement with previous research [40]. The combination with other CNS depressant drugs increases respiratory depression [15, 19] and the risk of fractures [20], and can be suggestive of substance abuse [19], as well as be detrimental on activities requiring alertness, i.e. driving.

This observational study has some limitations. Our analyses included data from two cross-sections of the Tromsø study; inferences on causality are difficult if not impossible due to the lack of temporality between exposure and effect.

The agreement between self-reported analgesic use and prescription records is moderate [41]. The rate of underreporting of self-reported use of ibuprofen and paracetamol is approximately 15 % or more [42]. Recall of NSAID use declines over time, and particularly among infrequent users of OTC NSAIDs [43]. Higher use of strong analgesics among non-participants compared to participants has been reported [9]. This all points toward an underestimation of the prevalence of analgesic use in the present study.

Participants reported the use of “painkillers”, leading to possible ambiguity and misclassification. We could not separate OTC and Rx use in the analyses on high-risk use. The contraindications and drug interactions identified may have been dealt with in an adequate manner by the prescribing physician or other health personnel. The most severe cases may have been missed due to non-participation, leading to an underestimation of high-risk analgesic use.

Study strengths include the use of a large, repeated population-based survey and self-reported data on both OTC analgesics and Rx analgesics. A comprehensive estimate of the use of analgesics cannot be done without the use of interview or questionnaires.

The Tromsø population may be regarded as representative of a Northern European, white, urban population [29], and the results may be generalizable to such populations.

## Conclusions

The use of analgesics increased in the time period, in line with other studies and gross sales statistics, mainly due to an increase in sporadic use of OTC analgesics. We have identified several areas of high risk use of analgesics where a known contraindication or drug interaction do not seem to lead to lower use at a population level. This could put people at risk and pose a threat to public health. Public and prescriber awareness about important contraindications, such as chronic kidney disease, gastrointestinal ulcers and risk of CVD, as well as clinically relevant drug interactions with analgesics, need to be increased.

## Abbreviations

ACE: Angiotensin-converting enzyme
ASA: Acetylsalicylic acid
ATC: Anatomical therapeutic chemical classification system
AT II: Angiotensin II
CI: Confidence interval
CKD-EPI: Chronic Kidney Disease Epidemiology collaboration
CNS: Central nervous system
COX-2: Cyclooxygenase 2
CVD: Cardiovascular disease
eGFR: Estimated glomerular filtration rate
GEE: Generalized estimating equations
NSAIDs: Non-steroidal anti-inflammatory drugs
OR: Odds ratio
OTC: “Over-the-counter”, non-prescription
Rx: Prescription
SSRI: Selective serotonin reuptake inhibitors

## References

[1] Association TNP (2012). Facts and figures - pharmacies and pharmaceuticals in Norway 2012. Oslo.

[2] Sakshaug S (2014). Drug consumption in Norway 2009–2013.

[3] Eggen AE (1993). The Tromso study: frequency and predicting factors of analgesic drug use in a free-living population (12–56 years). J Clin Epidemiol.

[4] Antonov K, Isacson D (1996). Use of analgesics in Sweden - the importance of sociodemographic factors, physical fitness, health and health-related factors, and working conditions. Soc Sci Med.

[5] Isacson D, Bingefors K (2002). Epidemiology of analgesic use: a gender perspective. Eur J Anaesthesiol.

[6] Paulose-Ram R, Hirsch R, Dillon C, Losonczy K, Cooper M, Ostchega Y (2003). Prescription and non-prescription analgesic use among the US adult population: results from the third National health and nutrition examination survey (NHANES III). Pharmacoepidemiol Drug Saf.

[7] Porteous T, Bond C, Hannaford P, Sinclair H (2005). How and why are non–prescription analgesics used in Scotland?. Fam Pract.

[8] Hargreave M, Andersen TV, Nielsen A, Munk C, Liaw KL, Kjaer SK (2010). Factors associated with a continuous regular analgesic use - a population-based study of more than 45,000 Danish women and men 18–45 years of age. Pharmacoepidemiol Drug Saf.

[9] Eggen AE (1996). The use of controlled analgesics in a general population (15–59 years)- the influence of age, gender, morbidity, lifestyle and sociodemographic factors. Pharmacoepidemiol Drug Saf.

[10] Antonov KI, Isacson DG (1998). Prescription and nonprescription analgesic use in Sweden. Ann Pharmacother.

[11] Saedder E, Brock B, Nielsen L, Bonnerup D, Lisby M (2014). Identifying high-risk medication: a systematic literature review. Eur J Clin Pharmacol.

[12] Schjerning Olsen A-M, Fosbøl EL, Gislason GH (2014). The impact of NSAID treatment on cardiovascular risk – insight from Danish observational data. Basic Clin Pharmacol Toxicol.

[13] de Abajo FJ, Gil MJ, Garcia Poza P, Bryant V, Oliva B, Timoner J, et al. Risk of nonfatal acute myocardial infarction associated with non-steroidal antiinflammatory drugs, non-narcotic analgesics and other drugs used in osteoarthritis: a nested case-control study. Pharmacoepidemiol Drug Saf. 2014;23(11):1128–38. doi:10.1002/pds.3617. 10.1002/pds.3617 24692325

[14] Schjerning Olsen A-M, Fosbøl EL, Lindhardsen J, Folke F, Charlot M, Selmer C, et al. Duration of treatment with nonsteroidal anti-inflammatory drugs and impact on risk of death and recurrent myocardial infarction in patients with prior myocardial infarction: a nationwide cohort study. Circulation. 2011;123(20):2226–35. doi:10.1161/circulationaha.110.004671. 10.1161/CIRCULATIONAHA.110.004671 21555710

[15] Aronson JK (2010). Meyler’s side effects of analgesics and anti-inflammatory drugs.

[16] Huerta C, Castellsague J, Varas-Lorenzo C, García Rodríguez LA (2005). Nonsteroidal anti-inflammatory drugs and risk of ARF in the general population. Am J Kidney Dis.

[17] Gooch K, Culleton BF, Manns BJ, Zhang J, Alfonso H, Tonelli M, et al. NSAID use and progression of chronic kidney disease. Am J Med. 2007;120(3):280e1–e7. doi:10.1016/j.amjmed.2006.02.015. 10.1016/j.amjmed.2006.02.015 17349452

[18] Baxter K, Preston C, editors. Stockley’s drug interactions. [online] London: Pharmaceutical Press; 2014

[19] Gudin JA, Mogali S, Jones JD, Comer SD (2013). Risks, management, and monitoring of combination opioid, benzodiazepines, and/or alcohol use. Postgrad Med.

[20] Li L, Setoguchi S, Cabral H, Jick S (2013). Opioid use for noncancer pain and risk of fracture in adults: a nested case-control study using the General practice research database. Am J Epidemiol.

[21] Tarantino G, Di Minno MND, Capone D (2009). Drug-induced liver injury: is it somehow foreseeable?. World J Gastroenterol.

[22] Roberts E, Delgado Nunes V, Buckner S, Latchem S, Constanti M, Miller P, et al. Paracetamol: not as safe as we thought? A systematic literature review of observational studies. Ann Rheum Dis. 2015. doi:10.1136/annrheumdis-2014-206914. 10.1136/annrheumdis-2014-206914 PMC4789700 25732175

[23] Adams R, Appleton S, Gill T, Taylor A, Wilson D, Hill C (2011). Cause for concern in the use of non-steroidal anti-inflammatory medications in the community - a population-based study. BMC Fam Pract.

[24] Koffeman AR, Valkhoff VE, Çelik S, Jong GW, Sturkenboom MC, Bindels PJ, et al. High-risk use of over-the-counter non-steroidal anti-inflammatory drugs: a population-based cross-sectional study. Br J Gen Pract. 2014;64(621):e191–8. doi:10.3399/bjgp14X677815. 10.3399/bjgp14X677815 PMC3964463 24686883

[25] Silvani MC, Motola D, Poluzzi E, Bottoni A, De Ponti F, Vaccheri A, et al. Gastro-intestinal problems and concomitant medication in NSAID users: additional findings from a questionnaire-based survey in Italy. Eur J Clin Pharmacol. 2006;62(3):235–41. doi:10.1007/s00228-005-0078-7. 10.1007/s00228-005-0078-7 16416304

[26] Stosic R, Dunagan F, Palmer H, Fowler T, Adams I (2011). Responsible self-medication: perceived risks and benefits of over-the-counter analgesic use. Int J Pharm Pract.

[27] Wilcox CM, Cryer B, Triadafilopoulos G (2005). Patterns of use and public perception of over-the-counter pain relievers: focus on nonsteroidal antiinflammatory drugs. J Rheumatol.

[28] Jacobsen BK, Eggen AE, Mathiesen EB, Wilsgaard T, Njolstad I (2012). Cohort profile: the Tromso study. Int J Epidemiol.

[29] Eggen AE, Mathiesen EB, Wilsgaard T, Jacobsen BK, Njølstad I (2013). The sixth survey of the Tromsø study (Tromsø 6) in 2007–08: collaborative research in the interface between clinical medicine and epidemiology: study objectives, design, data collection procedures, and attendance in a multipurpose population-based health survey. Scand J Public Health.

[30] Hallan SI, Coresh J, Astor BC, Asberg A, Powe NR, Romundstad S, et al. International comparison of the relationship of chronic kidney disease prevalence and ESRD risk. J Am Soc Nephrol. 2006;17(8):2275–84. doi:10.1681/asn.2005121273. 10.1681/ASN.2005121273 16790511

[31] Levey AS, Stevens LA, Schmid CH, Zhang Y, Castro IIIAF, Feldman HI, et al. A new equation to estimate glomerular filtration rate. Ann Intern Med. 2009;150(9):604–12. doi:10.7326/0003-4819-150-9-200905050-00006. 10.7326/0003-4819-150-9-200905050-00006 PMC2763564 19414839

[32] Selmer R, Lindman AS, Tverdal A, Pedersen JI, Njolstad I, Veierod MB (2008). Model for estimation of cardiovascular risk in Norway. Tidsskr Nor Laegeforen.

[33] The Norwegian Directorate of Health. National guideline on individual primary prevention of cardiovascular disease. 2009. Available at: http://www.helsedirektoratet.no/.

[34] Statistics Norway. Population, by sex and age (table 10211). Available at: http://www.ssb.no/.

[35] Fredheim OM, Skurtveit S, Breivik H, Borchgrevink PC (2010). Increasing use of opioids from 2004 to 2007 - pharmacoepidemiological data from a complete national prescription database in Norway. Eur J Pain.

[36] Hamunen K, Paakkari P, Kalso E (2009). Trends in opioid consumption in the Nordic countries 2002-2006. Eur J Pain.

[37] Ruscitto A, Smith BH, Guthrie B (2014). Changes in opioid and other analgesic use 1995–2010: Repeated cross-sectional analysis of dispensed prescribing for a large geographical population in Scotland. Eur J Pain.

[38] Carlsten A, Wennberg M, Bergendal L (1996). The influence of Rx-to-OTC changes on drug sales. Experiences from Sweden 1980–1994. J Clin Pharm Ther.

[39] Plantinga L, Grubbs V, Sarkar U, Hsu CY, Hedgeman E, Robinson B, et al. Nonsteroidal anti-inflammatory drug use among persons with chronic kidney disease in the United States. Ann Fam Med. 2011;9(5):423–30. doi:10.1370/afm.1302. 10.1370/afm.1302 PMC3185478 21911761

[40] Mellbye A, Svendsen K, Borchgrevink PC, Skurtveit S, Fredheim OMS (2012). Concomitant medication among persistent opioid users with chronic non-malignant pain. Acta Anaesthesiol Scand.

[41] Nielsen MW, Søndergaard B, Kjøller M, Hansen EH (2008). Agreement between self-reported data on medicine use and prescription records vary according to method of analysis and therapeutic group. J Clin Epidemiol.

[42] Loo RL, Chan Q, Brown IJ, Robertson CE, Stamler J, Nicholson JK, et al. A comparison of self-reported analgesic use and detection of urinary ibuprofen and acetaminophen metabolites by means of metabonomics: The INTERMAP study. Am J Epidemiol. 2012;175(4):348–58. doi:10.1093/aje/kwr292. 10.1093/aje/kwr292 PMC3271812 22223708

[43] Lewis JD, Strom BL, Kimmel SE, Farrar J, Metz DC, Brensinger C, et al. Predictors of recall of over-the-counter and prescription non-steroidal anti-inflammatory drug exposure. Pharmacoepidemiol Drug Saf. 2006;15(1):39–45. doi:10.1002/pds.1134. 10.1002/pds.1134 16136614

