# Erratum to: Analgesic use in a Norwegian general population: change over time and high-risk use - The Tromsø Study

**DOI:** 10.1186/s40360-017-0158-1

**Published:** 2017-06-27

**Authors:** Per-Jostein Samuelsen, Lars Slørdal, Ulla Dorte Mathisen, Anne Elise Eggen

**Affiliations:** 1 Regional Medicines Information and Pharmacovigilance Centre (RELIS), University Hospital of North Norway, N-9038 Tromsø, Norway; 2 Department of Community Medicine, UiT-The Arctic University of Norway, Tromsø, Norway; 3 Department of Laboratory Medicine, Children’s and Women’s Health, Norwegian University of Science and Technology, Trondheim, Norway; 4 Department of Clinical Pharmacology, St. Olav University Hospital, Trondheim, Norway; 5 Section of Nephrology, University Hospital of North Norway, Tromsø, Norway

## Erratum

Upon publication of the original article [1], it was noticed that the following reference was erroneously left out:

Ademi Z, Turunen JH, Kauhanen J, Enlund H (2007) A comparison of three questionnaire-based measures of analgesic use over 11 years in adult males: a retrospective analysis of data from a prospective, population-based cohort study. Clin Ther 29 (3): 529–534

The reference should have been included after the sentence: “A comprehensive estimate of the use of analgesics cannot be done without the use of interview or questionnaires.” found towards the end of the Discussion section on page eight, second column, paragraph six, second sentence.
